# Pan-tropical Sentinel-2 cloud-free annual composite datasets

**DOI:** 10.1016/j.dib.2021.107488

**Published:** 2021-10-18

**Authors:** D. Simonetti, U. Pimple, A. Langner, A. Marelli

**Affiliations:** a European Commission, Joint Research Centre, Ispra, VA 21027, Italy; b The Joint Graduate School of Energy and Environment (JGSEE) and Centre of Excellence on Energy Technology and Environment, King Mongkut's University of Technology Thonburi, Bangkok 10140, Thailand; c Arcadia SIT for European Commission, Joint Research Centre, Ispra, VA 21027, Italy

**Keywords:** Copernicus Sentinel-2, cloud free composite, cloud mask, pixel-based classification, BRDF correction, annual change

## Abstract

Sentinel-2 MSI is one of the core missions of the Copernicus Earth Observation programme of the European Union. This mission shows great potential to map the regional high-resolution spatio-temporal dynamics of land use and land cover. In tropical regions, despite the high revisiting time of 5 days including both Sentinel-2A and 2B satellites, the frequent presence of clouds, cloud-shadows, haze and other atmospheric contaminants are precluding the visibility of the Earth surface up to several months. In this paper we present four annual pan-tropical cloud-free composites computed and exported from Google Earth Engine (GEE) by making use of available Sentinel-2 L1C collection for the period spanning from 2015 to 2020. We furthermore propose empirical approaches to reduce the BRDF effect over tropical forest areas by showing pros and cons of image-based versus swath-based methodologies. Additionally, we provide a dedicated web-platform offering a fast and intuitive way to browse and explore the proposed annual composites as well as layers of potential annual changes as a ready-to-use means to visually identify and verify degradation and deforestation activities as well as other land cover changes.

## Specifications Table

**Table:** 

Subject	Earth and Planetary Sciences (General)
Specific subject area	Remote sensing, Big Data, Land Cover
Type of data	Satellite GeoTiff images with 3 bands at 20m resolution WMS tiles service
How data were acquired	Computed and exported from Google Earth Engine (GEE)
Data format	Analyzed and filtered Sentinel-2 L1C imagery. Secondary data are available as GeoTiff files via a dedicated web page and WMS service
Parameters for data collection	Annual median composite of all Sentinel-2 raw images acquired in 2015-2017, 2018, 2019, 2020
Description of data collection	Clouds and shadows masking and annual median composite of Sentinel-2 L1C imagery as available in Google Earth Engine (GEE). Empirical BRDF correction. Annual change layers.
Data source location	Secondary data spatial extent (WGS 84, EPSG: 4326): upper Left: 118 W, 38 N, lower Right: 156 E, 56 S Primary data: global Sentinel-2 L1C data covering all landmass as acquired by ESA and available in Google Earth Engine (GEE) cloud computing platform (https://earthengine.google.com)
Data accessibility	Dedicated website for data browsing and download: https://forobs.jrc.ec.europa.eu/recaredd/S2_composite.php WMS service to embed the proposed composite into ArcMap, QGis or any other websites GEE code for custom processing: https://code.earthengine.google.com/ae29b5a2a7ccaa0943ac521d5971603c Repository name: Joint Research Centre Data Catalogue doi: https://doi.org/10.2905/5EF706BB-CCED-47AA-B5F3-F56112B420C2 PID: http://data.europa.eu/89h/5ef706bb-cced-47aa-b5f3-f56112b420c2

## Value of the Data

• The annual cloud-free pan-tropical composites facilitate the use of Sentinel-2 Multispectral Instrument (MSI) Level 1C imagery for national forestry services in tropical countries, especially in relation to Reducing Emissions from Deforestation and Forest Degradation (REDD+) activities and the United Nations Restoration Declaration Decade (2021–2030) agenda on diversity restoration.
• Anyone aiming at large-scale land cover/land use change mapping (LCLUC) including photo-interpretation, automatic classification and validation can benefit from the pre-computed annual composites and change layer. In particular, the following stakeholder groups would be the main users: the European Commission, conservationists, policy makers, financiers, donors, international development agencies including national governments, stakeholders of the research and scientific community, non-governmental organizations (NGOs) as well as commercial companies.
• The annual composites can be used for automatic classification, feature extraction, visual inspection, validation, change detection, computation of NDVI, NBR, NDWI and any other spectral indexes based on RED, NIR and SWIR1 bands.
• The pre-computed composites derived from Sentinel-2 L1C imagery are accessible, free of charge, throughout a dedicated web-portal as well as via WMS services to be easily integrated into other websites or desktop GIS software. With a 20 m resolution in the RED, NIR, SWIR1 bands, geographic Lat-Lon WGS84 projection and 8 bit data type, each composite is a light weight product (e.g., only 200 GB for the entire Africa) easy to handle in both local and regional-scale mapping activities. GeoTiff tiles of 10°·10° can be eventually downloaded for local processing and analysis.

## 1 Data Description

### 1.1 Context and reference information

The Sentinel-2 mission of the European Copernicus Earth observation program started with the launch of the first satellite in June 2015 and became operational in late 2017 when the constellation of the two polar-orbiting satellites (2A and 2B) was able to acquire images of the land and coastal areas at a spatial resolution ranging from 10 to 60 m in the optical domain (13 spectral bands from 0.44–2.2 µm) and a revisit time of 5 days at the equator [1].

The above-mentioned characteristics, accompanied by a free, full and open access distribution policy, are making Sentinel-2 imagery a valuable product for LULCC mapping initiatives either at regional, national or continental scale.

The work described in this manuscript has been conducted in the framework of the ReCaREDD project (Reinforcement of Capacities for REDD+) [2], an international initiative aiming at enhancing the capacity of institutions in tropical partner countries to report on deforestation and forest degradation in a reliable and cost‐efficient manner.

**Fig. 1:**
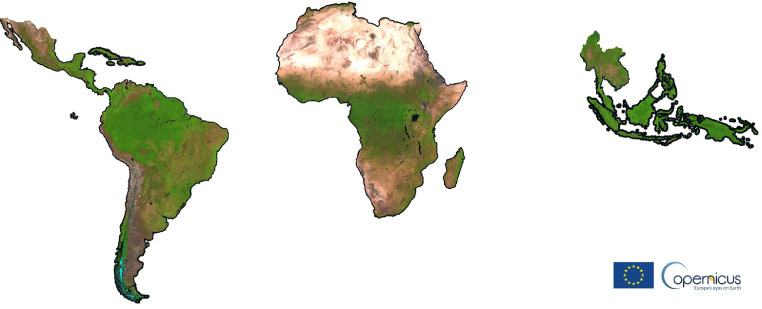
Extent of the proposed annual Sentinel-2 L1C cloud-free composites. False colour combination (SWIR1,NIR,RED) of year 2020 median layer.

Although applicable at global scale, the proposed methodology has been developed and applied only within the pan-tropical scope of the project (Fig. 1), where the persistent cloud cover, cloud shadows and haze frequently result in no exploitable images over large areas for weeks or even months. Hence the need for a robust methodology capable of removing any pixel affected by atmospheric contaminations and using the few available cloud-free ones to compute the annual synthesis.

In order to cope with the limited internet bandwidth and computing power of many tropical partner institutions, the proposed annual composites have been produced only for the SWIR1, NIR and RED bands, with a spatial resolution of 20 m, a geographic Lat-Long, WGS84 projection and 8-bit data type. This configuration reduces the original size of each 3 bands composite by a factor of 6 (0.5 TB vs 3 TB).

The Google Earth Engine (GEE) [3] script link available in the “data accessibility” section can be used to recompute and export a customized composite with any time frame, band combination, resolution and projection thanks to a dedicated and user-friendly graphical user interface.

### 1.2 Annual cloud-free composites

This article is proposing four Sentinel-2 L1C cloud-free pan-tropical annual composites for the period 2015–2020 by extracting per-band annual median values after cloud and shadow masking based on spectral conditions specifically developed for tropical regions (Fig. 1). This set of rules, further described in chapter 2.1, have been compiled in a library named PINO (convenient trade name, not acronym).

All available raw Sentinel-2A and 2B L1C Top of Atmosphere (TOA) Reflectance images have been processed in the GEE cloud computing platform and downloaded by selecting only SWIR1, NIR and RED bands converted to 8 bit (Byte) using a multiplicative factor of 0.051 for visualization purposes and size optimization.

For the 2020 composite a post processing step, aiming at equalising the different acquisition swaths, has been applied as described in chapter 2.3.

Annual composites are accessible via the two following options:

• a dedicated website for fast browsing and download: https://forobs.jrc.ec.europa.eu/recaredd/S2_composite.php
• WMS service to visualize composite layers in QGis, ArcMap, IMPACT Toolbox as well as any other web-gis site: https://ies-ows.jrc.ec.europa.eu/iforce/sentinel2_composite/wms.py?

Due to the scarcity of available images at the beginning of the Sentinel-2 missions (Sentinel 2A was launched in June 2015 while 2B in March 2017), the first proposed composite covers a time frame spanning from 2015 to 2017.

### 1.3 Potential annual change layers

**Fig. 2:**
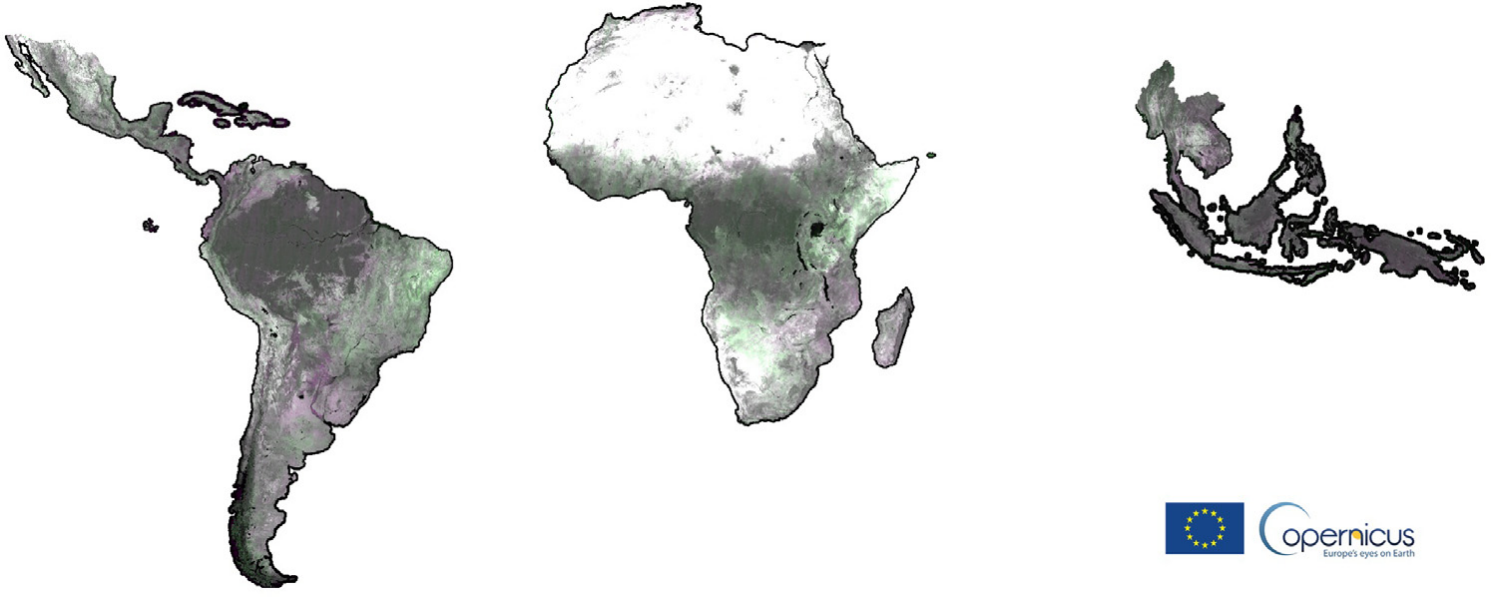
Extent of 2019-2020 potential change layer in a false colour combination (SWIR1^20^, SWIR1^19^, SWIR1^20^).

Additional layers, showing potential annual land cover changes, are computed and visualized on the fly using the SWIR1 bands of two selected GeoTiff yearly composites (Y1, Y2) (Fig. 2) in a false colour combination virtual file (vrt) as follows R: SWIR1^Y2^, G: SWIR1^Y1^, B: SWIR1^Y2^. This visualization choice and technique does not need any image pre-processing and highlights the loss and gain of photosynthetic activity on the ground in purple and green tones respectively as the direct consequence of the absorption (vegetation) or reflection (soil) in this portion of the electromagnetic spectrum.

**Fig. 3:**
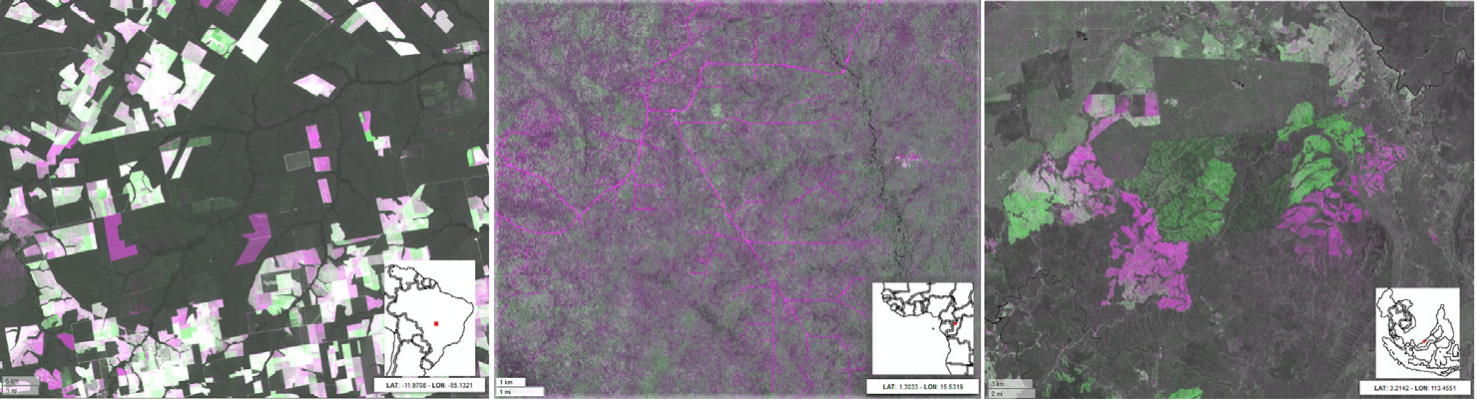
Change layers; from left: forest management in Mato Grosso (Brazil), road network in Sanga (Congo), new plantation in Sarawak state (Malaysia). The false colour composite based on the SWIR1^Y2^, SWIR1^Y1^, SWIR1^Y2^ band combination shows the loss of vegetation (higher spectral response in the SWIR1^Y2^ band) in purple while in green, the increase in photosynthetic activity hence, an absorption in the SWIR1^Y2^ band.

The intensity of the original SWIR1 reflectance band is preserved when no changes are occurring leading to dark grey tones in dense moist forest or bright ones in arid regions. Despite being sensitive to residual cloud and shadow contaminations in the reference composites, the change layers may serve as a ready-to-use means to visually identify and verify land cover changes, as well as degradation processes (Fig. 3).

### 1.4 Download

**Fig. 4:**
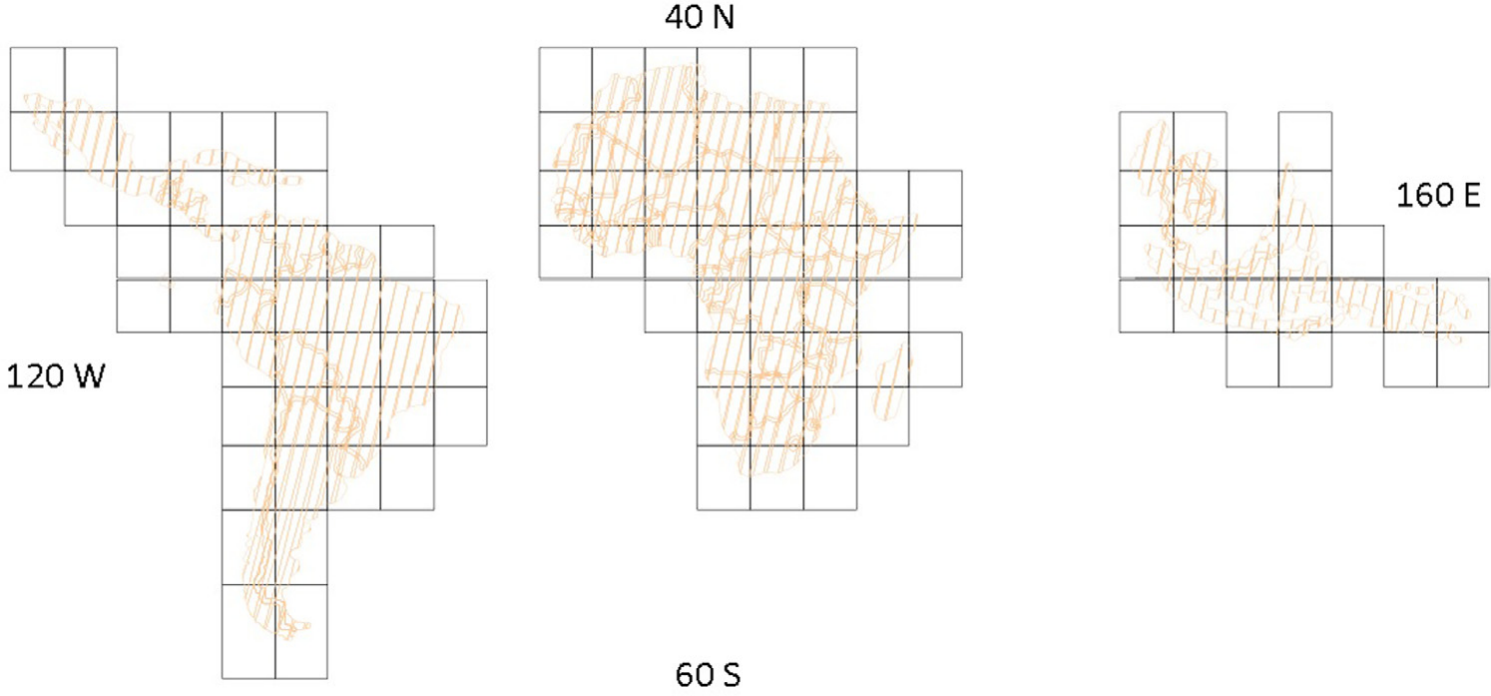
Extent and location of individual downloadable tiles.

Each annual pan-tropical composite is composed of 10°x10° tiles, corresponding to 112 downloadable GeoTiff files (Fig. 4) with internal cubic overviews and a total size of approximately 500 GB.

Each file is identified by a prefix corresponding to the central coordinates of the box, the continent, the year and the band order e.g., N35_W115_LAC_composite_2019_1184.tif corresponds to the upper left tile in Latin America and Caribbean region. Other continent identifiers are AFR (Africa) and SEA (Southeast Asia). The proposed gridding system, although leading to multiple downloads for large countries, reduces the volume of data to be transferred by single request to better cope with internet bandwidth and connection failures as reported by several stakeholders operating in the tropical belt.

The GEE script provided in the ‘data accessibility’ section can be eventually used to export a customized version of the composite.

## 2 Experimental Design, Materials and Methods

The new Sentinel-2 Global Mosaic service (S2GM) [4] of the Copernicus Global Land Service is providing on-demand composites from Sentinel-2 surface reflectance (level 2A). However, the algorithms used for the image compositing rely on the scene classification of Sentinel-2 L2A data, which is prone to errors (e.g., confusion between clouds, bright soil and built-up areas) eventually resulting in undesired artefacts in the S2GM products [5].

**Fig. 5:**
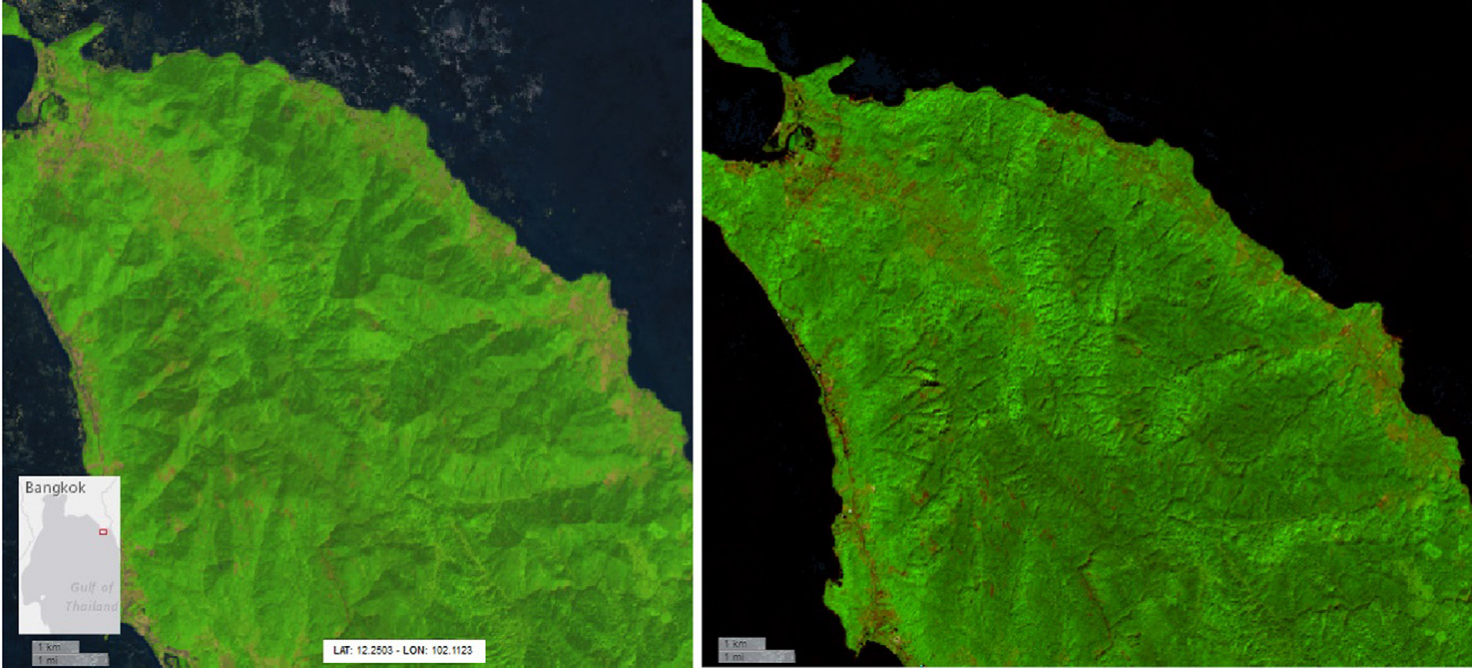
Ko Chang Island, Thailand, Sentinel-2 L1C TOA reflectance 2019 annual median composite processed in GEE (left) and overcorrection of north-facing slopes as obtained from S2GM 2019 annual composite (right) using the L2A BOA reflectance products. Both images are displayed in false colour composite (SWIR1, NIR, RED).

The L2A level, Bottom of the Atmosphere (BOA) reflectance, should ensure a bidirectional reflectance distribution function (BRDF) corrected product, hence uniform in space and time. However, the L2A products currently produced by the European Space Agency (ESA) Copernicus Ground Segment with the Sen2Cor processor [6] does not fully resolve the differences in brightness across the acquisitions swath in west-east direction and suffer from overcorrection over north-facing slopes as visible on Fig. 5.

**Fig. 6:**
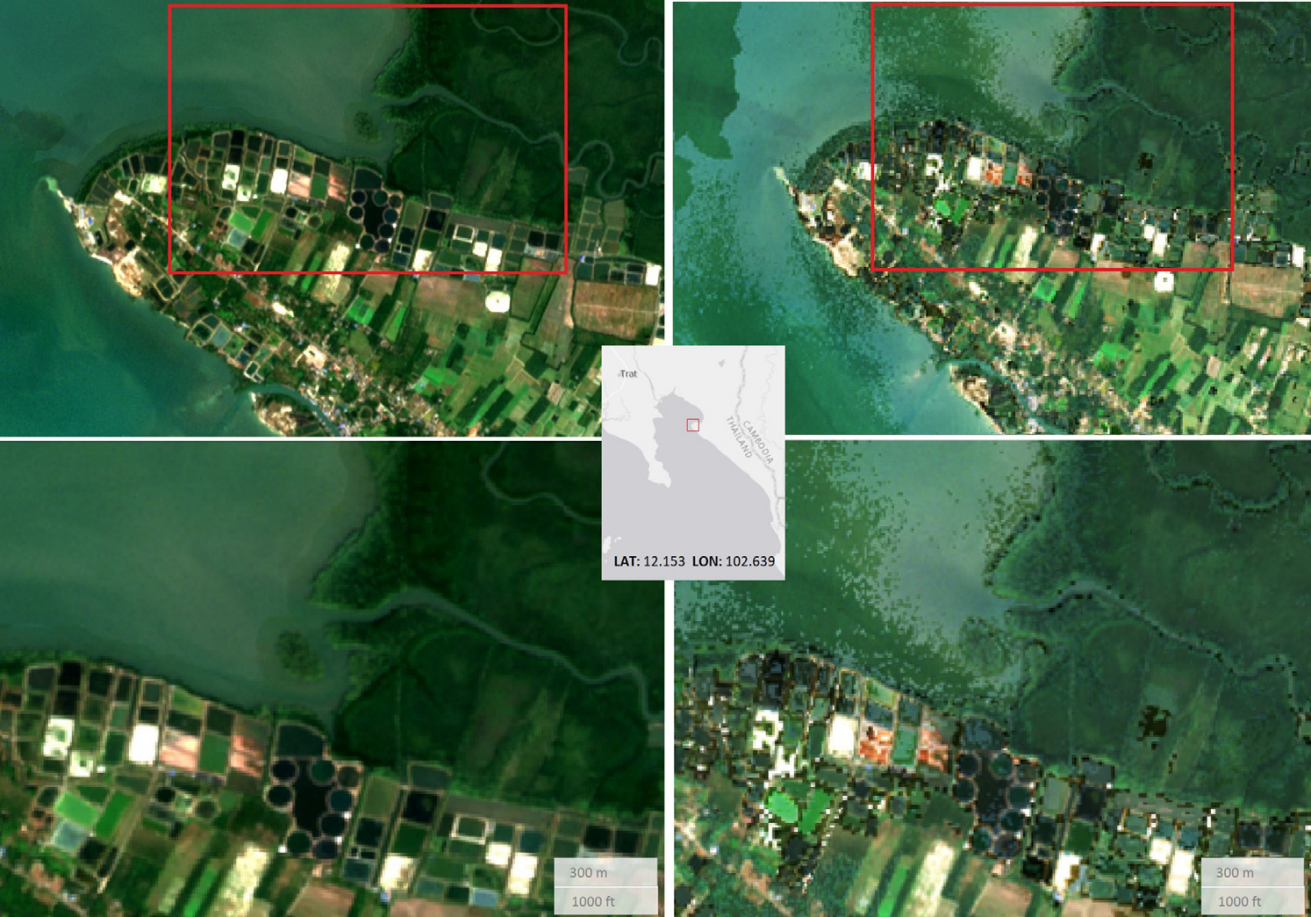
Trat province of Thailand, January 2019 true colour composite at 10 m resolution (overview and zoom) as computed in GEE using L2A median value (left) and by S2GM using L2A Medoid/STC algorithm (right).

Additionally, the best pixel Medoid/STC-S2 mosaicking algorithms [7] preserve the spectral profile of each pixel but introduces spatial heterogeneity also referred to as the “salt and pepper” effect as demonstrated on Fig. 6. In order to cope with that problematic, Copernicus is further developing Sen2Cor and the S2GM service is working on alternative compositing algorithm (ArtCom [8]) based on mean values, providing spatially smoother and visually more appealing products.

**Fig. 7:**
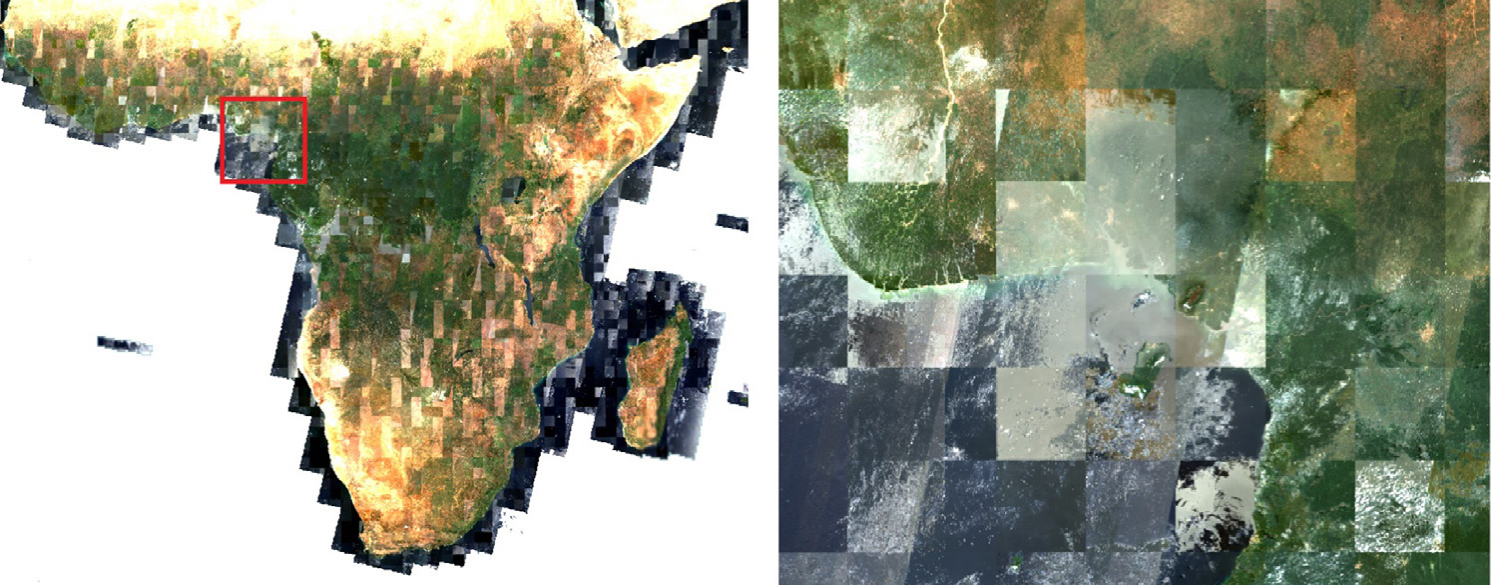
Africa Sentinel-2 L1C 2017 true colour (R, G, B) annual composite as proposed by Kempeneers et al. and zoom over Nigeria.

Kempeneers et al. [9] proposed an image compositing based on quick looks and the cloud mask of Level 1C original images but resulted in a global composite contaminated with persistent cloud coverage and tiling effect limiting its usage for large scale classification purposes (Fig. 7).

**Fig. 8:**
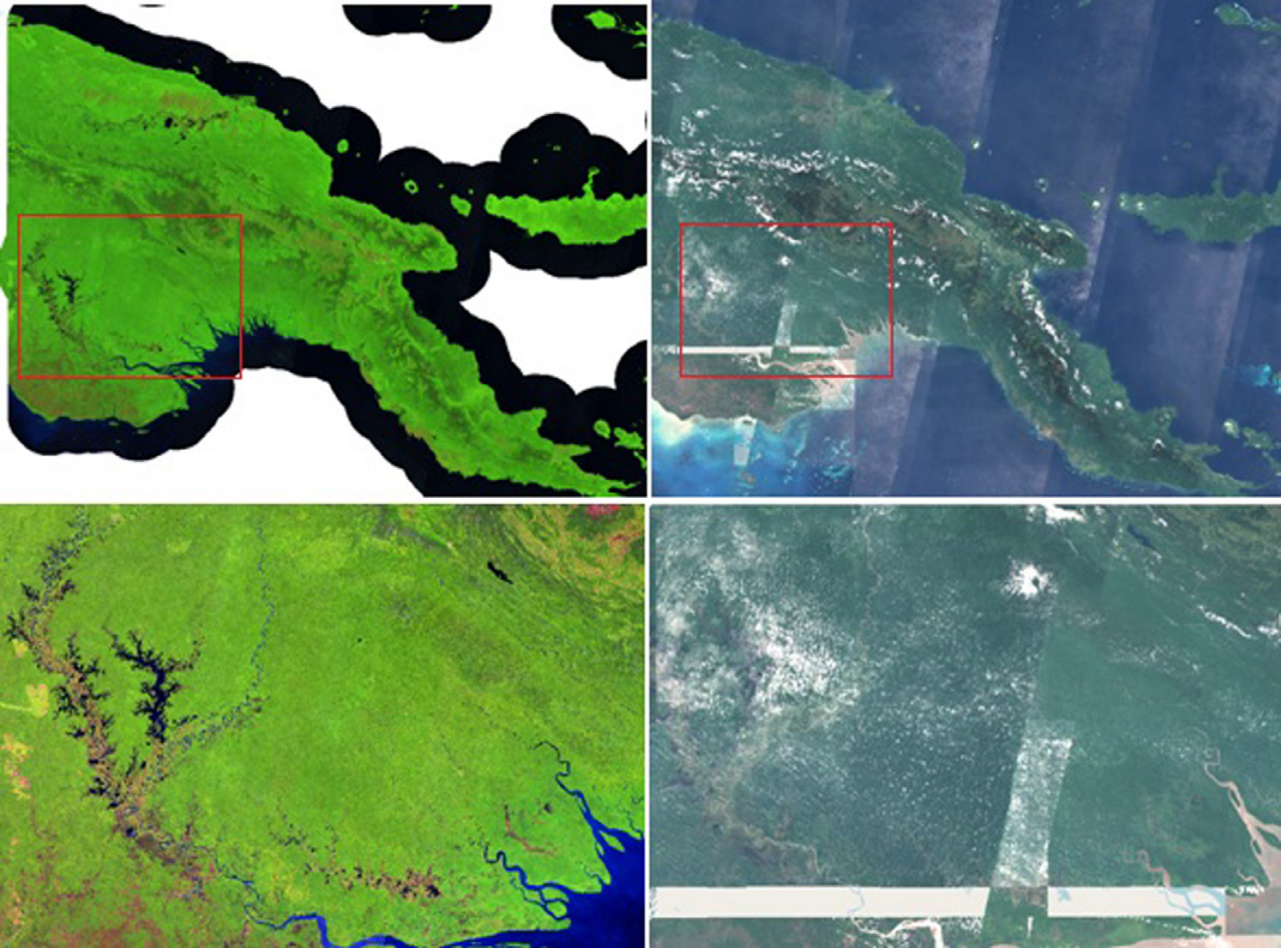
Sentinel-2 L1C annual composite over Papua New Guinea (PNG) using different compositing approaches: the proposed 2018 median composite in false colour (SWIR1, NIR, RED) on the left and the 25th percentile of 2017-2018 from Corbane et al. in true colour composite on the right.

Corbane et al. [10] proposed a compositing approach by computing the distribution of all selected observations in a UTM grid zone with the 25^th^ percentile of least cloudy pixels. Although the reference year has been set to 2018, image selection was extended to 2017 in cloudy areas (cloud percentage above 30%) in order to avoid data gaps (Fig. 8). This approach increased the likelihood to use the pixels stemming from the same season, hence reducing phenology changes within the same UTM. The tiling effect has been drastically reduced but acquisition swaths are still pronounced on the final product. This global composite is available at 10 m resolution for the RED, GREEN BLUE and NIR bands.

The quality of the above-mentioned composites is strongly related to the quality of the official Sentinel-2 L1C and L2A cloud masks provided by the Copernicus service: although good enough for less cloudy areas, the frequent cloud cover in tropical regions often compromises the quality of the final composite products.

Several ready to use cloud and cloud-shadow masking algorithms such as the Automated Cloud-Cover Assessment Algorithm (ACCA) [11], F-mask [12] and Hollstein et al. [13] have been tested. However, due to the need of local fine-tuning, application of morphological filters to incorporate the cloud edge, computing time restrictions and overall quality, these algorithms could not be efficiently applied over larger cloudy areas.

**Fig. 9:**
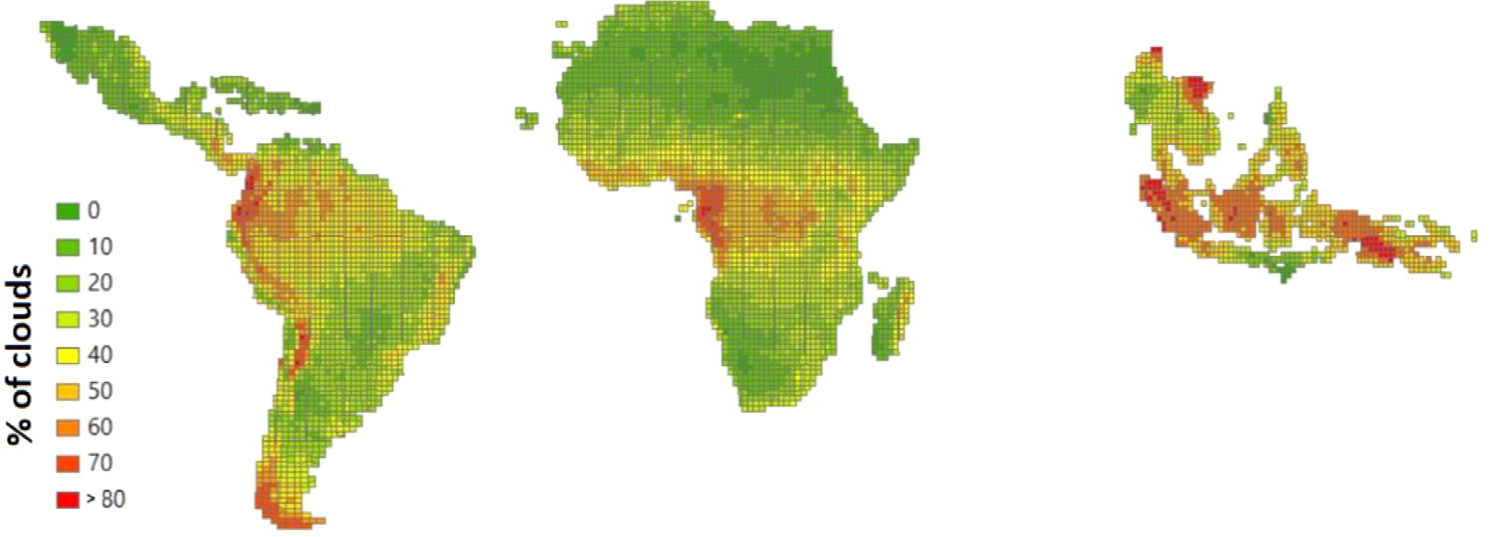
Average cloud cover percentage in year 2020 per MGRS tiles as extracted from image metadata.

The algorithm adopted for clouds and shadows masking in the proposed annual composites, namely PINO (described in section 2.1), is the evolution of the threshold-based rule-set proposed by Simonetti et al. [14]. Originally developed to operate only with Landsat TM/ETM/OLI sensors at a spatial resolution of 30 m, the rule-set has been further developed adding a decision tree tailored to the spatial and spectral characteristic of the Sentinel-2 imagery so as to better detect small and sparse clouds over the tropical belt where the annual average cloud percentage computed over the Military Grid Reference System (MGRS) tile [15] can be greater than 80% of the total observations (Fig. 9) hence limiting the possibility of sensing cloud-free images.

**Fig. 10:**
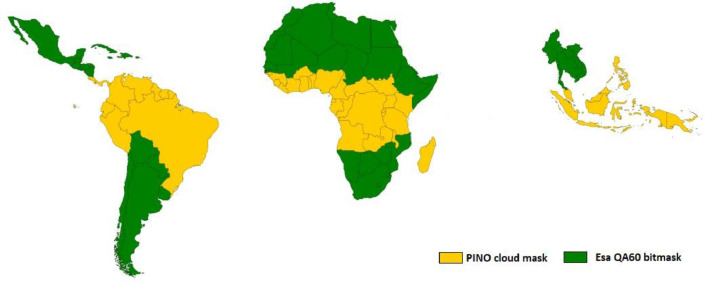
Cloud mask algorithm used in 2020 composite: proposed PINO in yellow, ESA bitmask in green.

The PINO algorithm has been applied to cloud-prone countries, as highlighted in yellow in Fig. 10, while a simple mask based on QA60 band equal or greater than 1024 was sufficient in areas (green) with abundance of cloud free images (greater than 60% of total observations). The former approach is resource (CPU, RAM) demanding hence almost two times slower; however, the average execution time in GEE (per orbit, per country) remains within the 2 h.

Additionally, the 2019 composite of Colombia, Gabon and Equatorial Guinea, has been processed in early 2020 with an extra 3D shadow casting model (based on sun elevation and azimuth parameters) with the aim of maximizing the detection of non-contaminated pixels in presence of “mackerel sky” [16], a sky condition characterized by the alternance of clouds, shadows and small clear gaps in between. However, due to the resource-intense 3D models often leading to processing errors, for the computation of the 2020 annual composite in early 2021, the PINO rule-set has been enriched (version 26 described in section 2.1) with spectral thresholds able to better identify shadows over the tropical moist forest and avoid shadow-casting models and buffers.

Due to the large volume of pixels to be classified and normalized over time and space, the composites are computed and exported from Google Earth Engine (GEE).

**Fig. 11:**
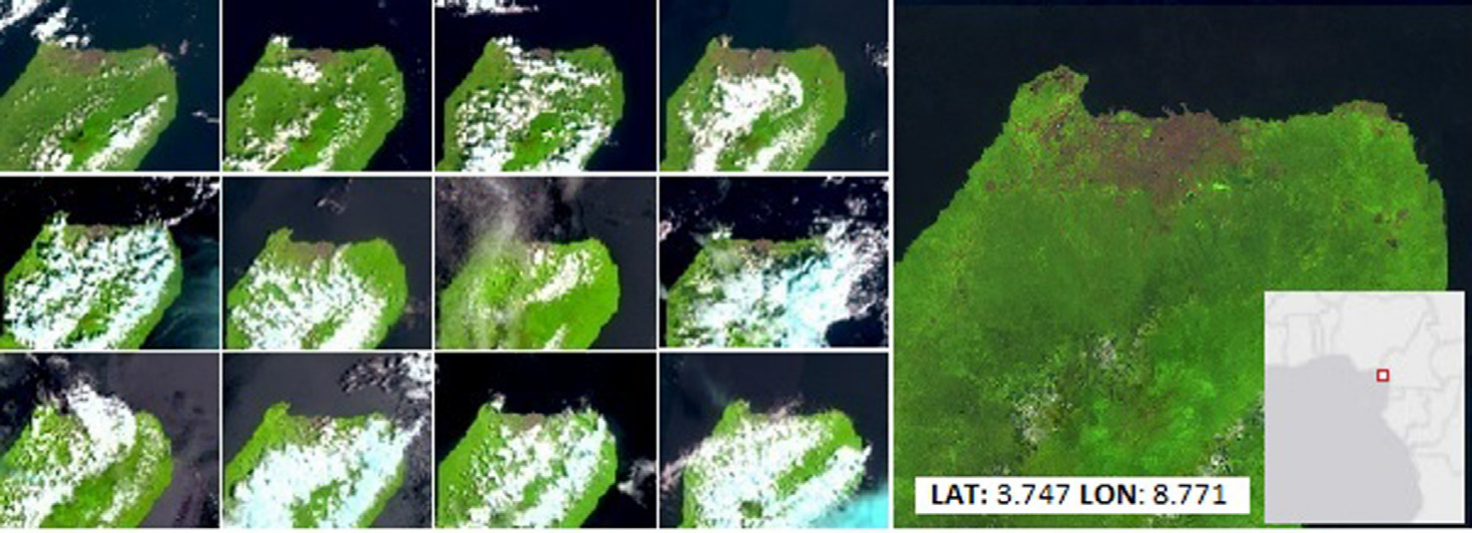
Best monthly Sentinel-2 acquisitions in 2019 over Malabo city, Bioko Island, Equatorial Guinea and the annual composite in false colour composite (SWIR1, NIR, RED).

Despite the fact that the proposed approach is resource (CPU, RAM) demanding, it allows creating high quality annual cloud-free composites in tropical areas affected by persistent cloud coverage such as in Malabo, Bioko Island (Equatorial Guinea), the cloudiest city in Africa [17]. The 12 least cloudy Sentinel-2 acquisitions in 2019 and the annual composite of all 68 available images corresponding to MGRS granules T32NMK, are displayed in Fig. 11.

The amount of contaminated pixels per single image prevents a wall-to-wall Land Cover-Land Cover Change (LC-LCC) mapping activity, hence the need for a pixel-based annual composite. However, in 2019 some forest areas of Bioko Island remained un-sensed by this optical sensor due to the fact that no single cloud free observation was available.

Please note that the median annual compositing approach i) does not preserves relationships between bands (Medoid always returns bands from the same selected pixel), ii) does not necessary catches the natural vegetation cycle and ii) can be strongly biased by the uneven distribution of cloud across the year, eventually leading to synthesis computed from images acquired in the dry season. However, by using the GEE script provided in the “data accessibility” section, new customized composites can be generated and exported to better cope with local or regional phenological conditions.

### 2.1 PINO classification: GEE code

The following code shows the JavaScript GEE implementation of the PINO cloud mask tailored to Sentinel-2 L1C images collected all over the tropical belt. Originally developed in 2014 by Simonetti et al. [14] to operate only with Landsat TM/ETM/OLI sensors at a spatial resolution of 30 m, it has been further developed adding decision tree tailored to the spatial and spectral characteristic of the Sentinel-2 imagery. The proposed release, version 26, does not apply any morphological filters (e.g., opening, closing) or buffering to cloud or shadow classes.

Spectral indices, as well as the median extraction, are based on TOA reflectance values hence subject to haze and other atmospheric contaminations. However, the cloud and shadow mask computed based on the L1C products as proposed hereafter, can be eventually applied to the correspondent Bottom of Atmosphere (BOA) reflectance image in the L2A collection.

#### 2.1.1 Decision tree approach

The classification algorithm relies on several spectral indexes (NDVI, NDSI, NDWI) as well as a set of fuzzy bands conditions (e.g., BLU > GREEN > RED) and fixed thresholds (e.g., B1 > 2000 and NDVI > 0.2) designed to recognize the spectral profile of different land cover types, clouds and shadows. This set of rules is the transposition in python code of the average profile of several spectral signatures, belonging to different classes, extracted from Sentinel-2 L1C images across the tropical belt.

After the initial cloud masking based on the QA60 band, the input image is dived into 3 broad thematic strata (1: water, 2: soil, 3: vegetation) on the basis of the NDVI values; within each stratum the spectral profile of each pixel is compared with the set of rules to identify clouds or shadows contaminations.

This approach allows i) better commission/omission error tracking in each stratum, ii) integrating tailored spectral rules, iii) distinguishing between clouds and shadows over water, soil or vegetation to apply further processing steps depending on the field of application of the composite (e.g., PINO has room of improvement in cloud/shadow detections over water).

The PINO function returns the same Sentinel-2 image received as input after replacing every pixel, classified as clouds or shadows, with no-data. Filtering the annual image collection before extracting the median value of each pixel, increases the probability of obtaining a cloud-free composite in cloud-prone areas or whenever the atmospheric contaminations affect more than 50% of the acquisitions.

// —- PINO Version 26 —;

var PINO = function(image){;

var BLU = image.select("B2");

var GREEN = image.select("B3");

var RED = image.select("B4");

var NIR = image.select("B8");

var NIRA = image.select("B8A");

var SWIR1 = image.select("B11");

var SWIR2 = image.select("B12");

var B1 = image.select("B1");

var B9 = image.select("B9");

var B10 = image.select("B10");

var QA60 = image.select("QA60");

var QA60gt0 = QA60.gt(0);

var th_NDVI_MAX_WATER = 0.0;

var th_NDVI_MIN_VEGE = 0.45;

var th_RANGELAND = 0.49;

var th_GRASS = 0.53;

var min123 = BLU.min(GREEN).min(RED);

var min1234 = min123.min(NIR);

var min234 = GREEN.min(RED).min(NIR);

var max234 = GREEN.max(RED).max(NIR);

var max1234 = max234.max(BLU);

var max57 = SWIR1.max(SWIR2);

var BLUgtGREEN = BLU.gt(GREEN);

var BLUlteNIR = BLU.lte(NIR);

var OUT = NIR.multiply(0);

var GREENgtRED = GREEN.gt(RED);

var GREENlteRED = GREEN.lte(RED.multiply(1.1));

var GREENgteRED = GREEN.gte(RED);

var REDlteNIR = RED.lte(NIRA);

var BLUsubtractNIR = BLU.subtract(NIR);

var BLUgtGREENgtRED = BLUgtGREEN.and(GREENgtRED);

var growing14 = (BLU.lte(GREEN)).and(GREENlteRED).and(REDlteNIR);

var growing15 = growing14.and(NIR.lte(SWIR1));

var decreasing123 = BLUgtGREEN.and(GREENgteRED);

var decreasing234 = (GREENgteRED).and(RED.gte(NIR));

var decreasing2345 = decreasing234.and(NIR.gte(SWIR2));

var NDVI = (NIR.subtract(RED)).divide(NIR.add(RED));

var NDWI = (GREEN.subtract(SWIR1)).divide(GREEN.add(SWIR1));

var WATERSHAPE= ((BLU.subtract(GREEN)).gt(-2000)).and(decreasing2345);

var ndvi_1 = NDVI.lte(th_NDVI_MAX_WATER);

var ndvi_2 = NDVI.lt(th_NDVI_MIN_VEGE).and(ndvi_1.eq(0));

var ndvi_3 = NDVI.gte(th_NDVI_MIN_VEGE);

var EsaMask = (QA60.eq(2048)).and (BLU.gt(1300)).and(B10.gt(150));

var cloudMask = (B1.gt(2000). and (B9.gt(400)));

      .or (B1.gt(2200). and (B9.gt(340)));

      .or (B1.gt(2200). and (B9.gt(280)). and (BLU.gt(2000)));

      .or (B1.gt(3000));

var SNOWSHAPE = ((min1234.gt(3000)). and (NDWI.gt(0.65)));

      .and (WATERSHAPE.eq(0)). and (QA60.eq(0));

// —– Classification section;

// SNOW;

OUT=OUT. where (SNOWSHAPE,100);

// LAVA FLOW;

OUT = OUT. where ((NDVI.lt(0.3)). and (max1234.lt(5000)). and (SWIR1.gt(10000)),110);

// MAIN CLOUDS;

OUT = OUT. where ((OUT.eq(0)). and (QA60.eq(2048). and (B1.gt(4000))),1);

OUT = OUT. where ((OUT.eq(0)). and (QA60.eq(1024). and (B1.gt(2400))),1);

OUT = OUT. where ((OUT.eq(0)). and (min1234.gt(2700)). and (B1.gt(2700)).and(B9.gt(300)),1);

OUT = OUT. where ((OUT.eq(0)). and (min1234.gt(2200)). and (B1.gt(2200)). and (B9.gt(500)),1);

OUT = OUT. where (cloudMask. and (NDVI.gt(0.12)),2);

OUT = OUT. where (OUT.eq(0). and (B1.gt(1850)). and (NDVI.gt(0.26)). and (RED.gt(1000)),2);

OUT = OUT. where (OUT.eq(0).and (QA60gt0). and (NIRA.gt(3000)). and (B1.gt(2500)),2);

OUT = OUT. where (OUT.eq(0). and (EsaMask.or(B10.gt(180))). and (B1.gt(1400)),2);

OUT = OUT. where (OUT.eq(0). and (QA60gt0). and (B1.gt(1500)). and (NIRA.gt(3500)). and (RED.gt(1000)),8);

OUT = OUT. where (OUT.eq(0). and (B1.gt(2000)). and (NDVI.gt(0.2)). and (BLU.gt(2000)). and (B9.gt(350)),2);

OUT = OUT. where (OUT.eq(1). and (QA60.eq(0)). and (B10.lt(50)). and (B9.lt(800)). and (NDVI.gt(-0.008)). and (growing15);

      . and (SWIR1.gt(NIRA)). and ((SWIR1.gt(4500)). and (B1.lt(2500));

      .or(SWIR1.gt(6000). and (B1.gt(4000)))),50);

// CLOUDS over WATER;

var CLOUDS_ON_WATER = OUT.eq(0). and (ndvi_1). and (decreasing2345). and (B1.gt(1800)). and (SWIR1.gt(700));

OUT = OUT. where (CLOUDS_ON_WATER. and (B9.gt(350)). and (B10.gt(15)),3);

OUT = OUT. where (CLOUDS_ON_WATER. and (SWIR1.gt(NIR)). and (B9.gt(150)),3);

OUT = OUT. where (OUT.eq(0). and (ndvi_1). and (decreasing2345). and (BLU.gt(1100)). and (;

    ((BLU.gt(1350)). and (B9.gt(350)). and (B10.gt(50)));

   .or((B1.gt(1550)). and (B9.gt(150)). and (SWIR1.gt(500)))),3);

OUT = OUT. where (OUT.eq(0). and (ndvi_1). and (B1.gt(2000)). and (SWIR1.gt(2000)),3);

OUT = OUT. where (OUT.eq(3). and (B10.lt(15)),50);

// SHADOWS on SOIL;

OUT = OUT. where (OUT.eq(0). and (ndvi_1). and (decreasing234). and (SWIR1.gt(400)),43);

OUT = OUT. where ((OUT.eq(0). and (ndvi_2). and (BLU.lt(1300)). and (BLUgtGREENgtRED). and (RED.lt(500))

      . and (BLUsubtractNIR.lt(1000))),41);

OUT = OUT. where ((OUT.eq(0). and (NDVI.lt(0.2). and (BLUgtGREENgtRED). and (RED.lt(800)). and (NIR.lt(900)). and (SWIR2.lt(200)))),37);

OUT = OUT. where (OUT.eq(37). and (NIRA.subtract(RED).gt(500)),40);

OUT = OUT. where (OUT.eq(0). and (ndvi_2). and (BLUgtGREENgtRED). and (;

      ((BLU.lt(1300)). and (RED.lt(600)). and (BLUsubtractNIR.lt(300)));

      .or((BLU.lt(1000)). and (RED.lt(500)). and (BLUsubtractNIR.lt(380)). and (B9.lt(100)))

      .or((NIR.subtract(GREEN).abs().lte(100)).add(BLUsubtractNIR.gte(100)). and (NIR.gte(600)). and (SWIR1.lt(500)))),41);

// SHADOWS on SOIL with NEG NDVI;

OUT = OUT. where (OUT.eq(0). and (NDVI.gt(-0.08)). and (WATERSHAPE). and (NIRA.gt(NIR)),41);

// SHADOWS on VEGETATION———————–;

OUT = OUT. where (NDVI.gt(0.4). and (RED.lt(350)). and (NIR.lt(2000)). and (SWIR2.lt(300)),40);

OUT = OUT. where (OUT.eq(41). and (NDVI.gt(0.4)),40);

var MyCOND = OUT.eq(0). and (ndvi_3). and (NDVI.lt(th_RANGELAND));

OUT = OUT. where (MyCOND. and (NIR.lt(1500)),40);

MyCOND = (ndvi_3). and (OUT.eq(0)). and (NDVI.lt(th_GRASS));

OUT = OUT. where (MyCOND. and (BLUlteNIR). and (NIR.lt(1400)),40);

OUT = OUT. where (MyCOND. and (BLU.gt(NIR)),40);

OUT = OUT. where (OUT.eq(0). and (B1.lt(1200)). and (NDVI.gt(0.6)). and (NDWI.gt(-0.3)). and (RED.lt(400)). and (B9.lt(300)). and (NIRA.lt(2500));

      . and (SWIR1.lt(850)),40);

OUT = OUT. where (((OUT.eq(0)). and (ndvi_1.not()). and (NDWI.lt(0.25)). and (;

   ((BLU.lt(1400)). and (BLU.gt(800)). and (BLUgtGREENgtRED). and (RED.lt(700)). and (NIR.lt(1450)). and (((NIR).subtract(BLU)).lt(300))))),41);

// BURNT or SOIL;

OUT = OUT. where ((OUT.eq(41)). and (ndvi_1.not()). and (SWIR2.gt(RED)). and (SWIR2.gt(600)),51);

MyCOND = OUT.eq(0). and (B1.gt(1200)). and (B9.gt(600)). and (RED.lt(1000));

OUT = OUT. where (MyCOND. and (B10.gt(100)). and (BLU.gt(1000)). and (;

   ((NIRA.gt(2000)). and (QA60gt0));

   .or((NIRA.gt(2300)). and (B9.gt(800)));

   .or((NIRA.gt(1800)). and (B9.gt(650)))),6);

OUT = OUT. where (MyCOND. and (BLU.gt(2000)). and (NIRA.gt(3500)). and (B10.gt(80)),6);

// SOIL TO CLOUDS;

OUT = OUT. where (OUT.eq(0). and (QA60gt0). and (B1.gt(1500)). and (RED.lt(1000)),6);

OUT = OUT. where (OUT.eq(55). and (QA60gt0). and (B1.gt(2000)),6);

OUT = OUT. where (OUT.eq(6). and (NDVI.gt(0.45)). and (RED.lt(900)). and (SWIR2.lt(1100)),60);

// EXTRA SHADOWS on VEGETATION;

OUT = OUT. where (OUT.eq(0). and (BLUgtGREENgtRED). and (NDVI.gt(0.3)). and (NDWI.gt(0)). and (B10.gt(45)),40);

OUT = OUT. where (OUT.eq(0). and (NDVI.gt(0.2)). and (NDWI.gt(0.1)). and (RED.lt(1000)),40);

OUT = OUT. where (OUT.eq(0). and (BLUgtGREENgtRED). and (NDVI.gt(0.2)). and (NDWI.gt(0)). and (B1.gt(1300)). and (RED.gt(800)). and (B9.gt(350));

      . and (B10.gt(45)). and (NIRA.gt(2000)). and (SWIR1.gt(1100)). and (SWIR2.gt(500)),40);

// THIN/OPAQUE CLOUD EDGE and SHADOWS on SOIL;

OUT = OUT. where (OUT.eq(0). and (B1.gt(1800)). and (BLUgtGREENgtRED). and (RED.gt(1000)). and (NIRA.gt(RED)). and (SWIR2.lt(RED)),6);

OUT = OUT. where (OUT.eq(0). and (QA60gt0). and (B1.gt(1400)). and (NDVI.gt(0.5)). and (B9.gt(500)). and (B10.gt(100)). and (NIRA.gt(2500));

      . and (SWIR1.gt(1500)),6);

OUT = OUT. where (OUT.eq(0). and (BLUgtGREENgtRED). and (QA60gt0). and (B10.gt(150)),6);

OUT = OUT. where (OUT.eq(41). and (NIR.gt(1200)). and (SWIR2.gt(350)),0);

OUT = OUT. where (OUT.eq(6). and (SWIR2.lt(600)). and (QA60gt0.not()),41);

OUT = OUT. where(OUT.eq(0). and (ndvi_2). and (decreasing123). and (SWIR2.lt(300)). and (RED.lt(1000)),40)

OUT = OUT. where(OUT.eq(0). and (NDVI.gt(0.40)). and (NDVI.lt(0.55)). and (decreasing123). and (SWIR2.lt(600)). and (RED.lt(600));

      . and (NIR.lt(2000)). and (SWIR1.lt(850)),40);

OUT = OUT. where ((OUT.eq(40)). and (SWIR1.gt(1000)),0);

// RECODING;

OUT = OUT. where (OUT.gte(50),0);

return image.updateMask (OUT.eq(0));

};

Median = S2collection. map(PINO). median();

### 2.2 Compositing by orbits

Despite the fact that double acquisitions provide a higher probability of getting cloud free observations in the overlapping area, pixels laying on the eastern and western side of the adjacent swaths present a considerable spectral difference caused by the BRDF effect, especially over the dense humid forest.

To overcome the heterogeneous spectral response in overlapping areas, the Sentinel-2 L1C 2019 and 2020 median composites have been processed by orbit and subsequently exported using the geographic Lat-Lon common projection instead of the multiple native UTM ones. Additionally, an inwards buffer of 8km has been applied to each swath (orbit) geometry to i) limit the amount of data to be processed and ii) remove sporadic detectors zig-zag along the edge of the scene and iii) guarantee a correct empirical BRDF correction as described hereafter.

**Fig. 12:**
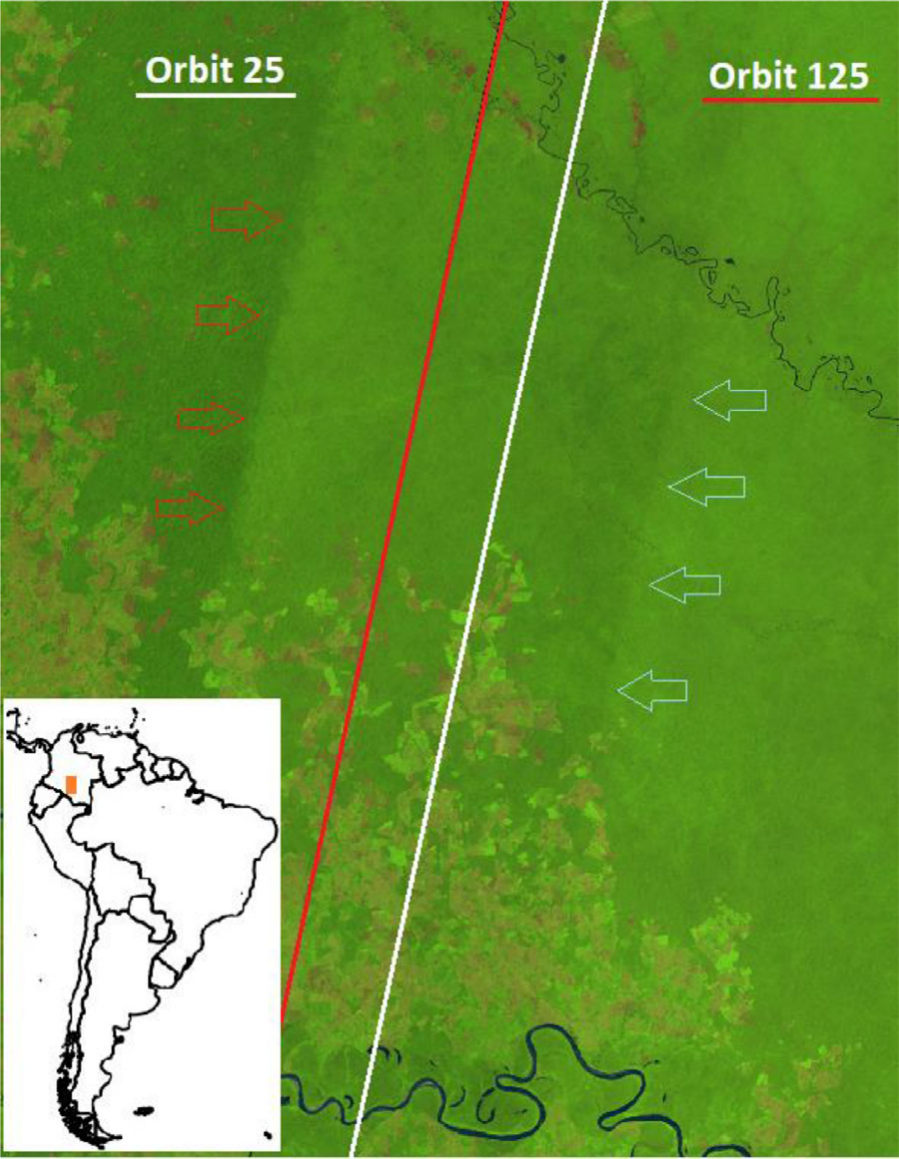
Colombia, 2018 false colour composite (SWIR1, NIR, RED) and the boundaries of the modified orbit number 125 (red) and 25 (white). Arrows indicate the edge of the original overlapping orbits, reflected in the annual composite (2018 has not been processed by orbit).

The red and white lines in Fig. 12 show the boundaries of the re-computed orbits number 125 and 25, respectively as available in the vector file [18] used in the compositing algorithm in 2019 and 2020; the 2018 median composite in background reflects the uneven distribution of images in the three distinct zones corresponding to the west orbit, the overlap with double acquisitions and east orbit.

### 2.3 Different empirical approaches to mitigate the BRDF effects on 2019 and 2020 composites

**Fig. 13:**
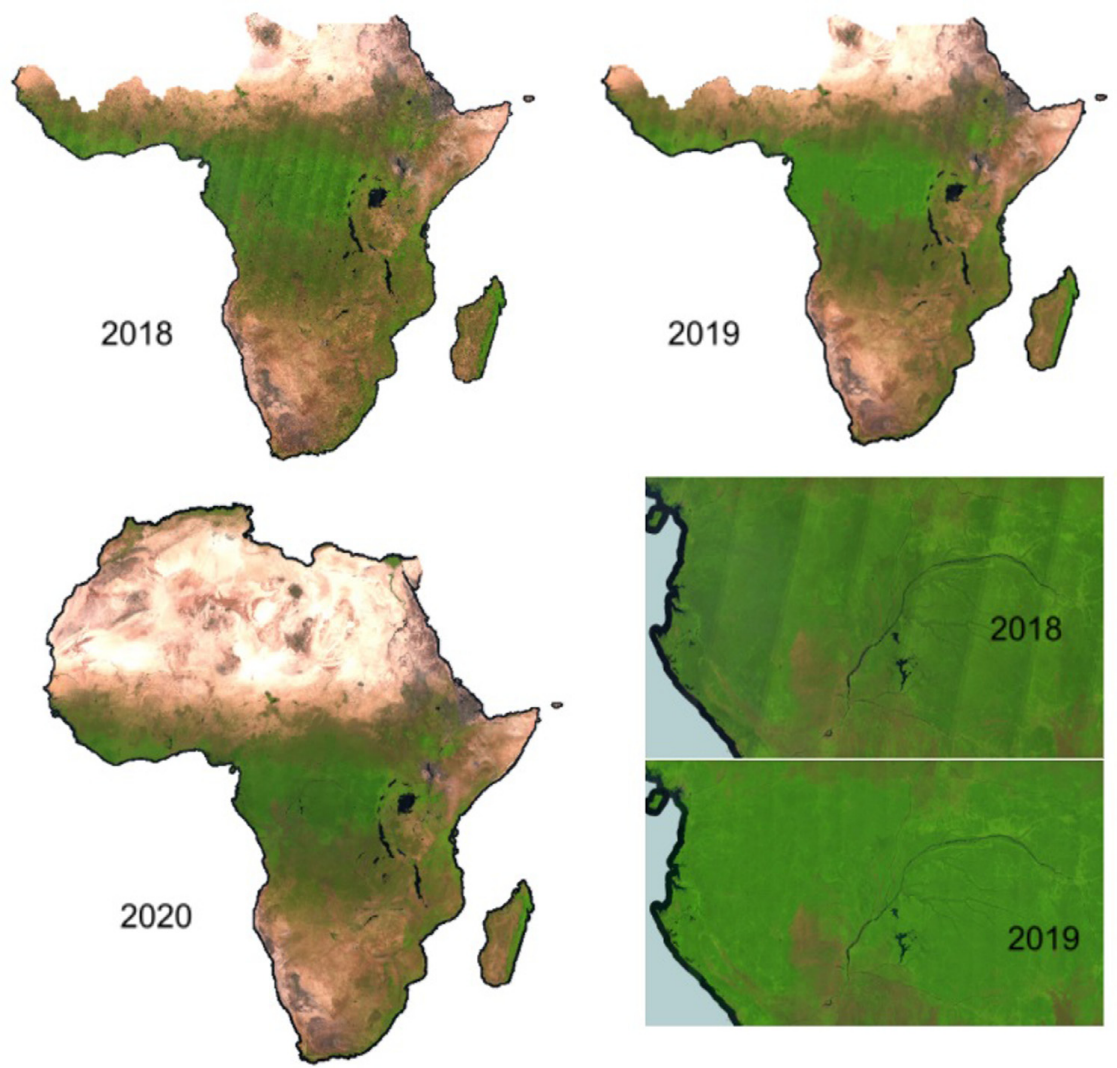
Different techniques to mitigate the radiometric non-uniformity affecting each orbit: raw data (2018), evergreen forest normalization of each MGRS tile (2019), linear gradient multiplier per orbit (2020). Composites are visualized in false colour (SWIR1, NIR, RED).

The Sentinel-2 2015-2017 and 2018 composites have been processed by computing the simple median value of each band across the year. However, the differences in brightness across the Sentinel-2 acquisitions in west-east direction, is visible in the final products, especially over the humid tropical forest as shown in Fig. 13.

**Fig. 14:**
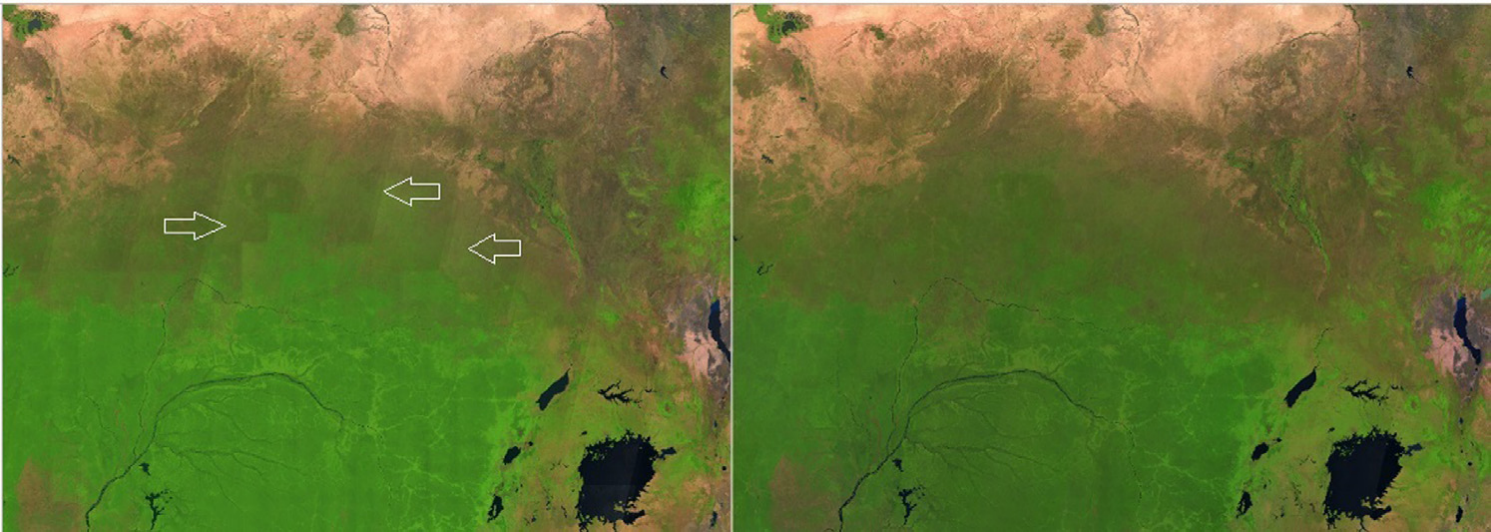
Central African Republic: visible tiling effect on the transition between dense forest and savannah when using the evergreen forest normalization at MGRS tile level (2019, left) and the smooth correction obtained with the orbit normalization approach (2020, right). Composites are visualized in false colour (SWIR1, NIR, RED).

In 2019, before computing the median value, each granule has been normalized using the dark object subtraction method (using evergreen forest as pseudo-invariant feature) with the aim of mitigating this BRDF radiometric effect. The evergreen forests in central Africa look homogeneous but tiles falling outside the evergreen domain, which are not normalized, are still affected (Fig. 14).

**Fig. 15:**
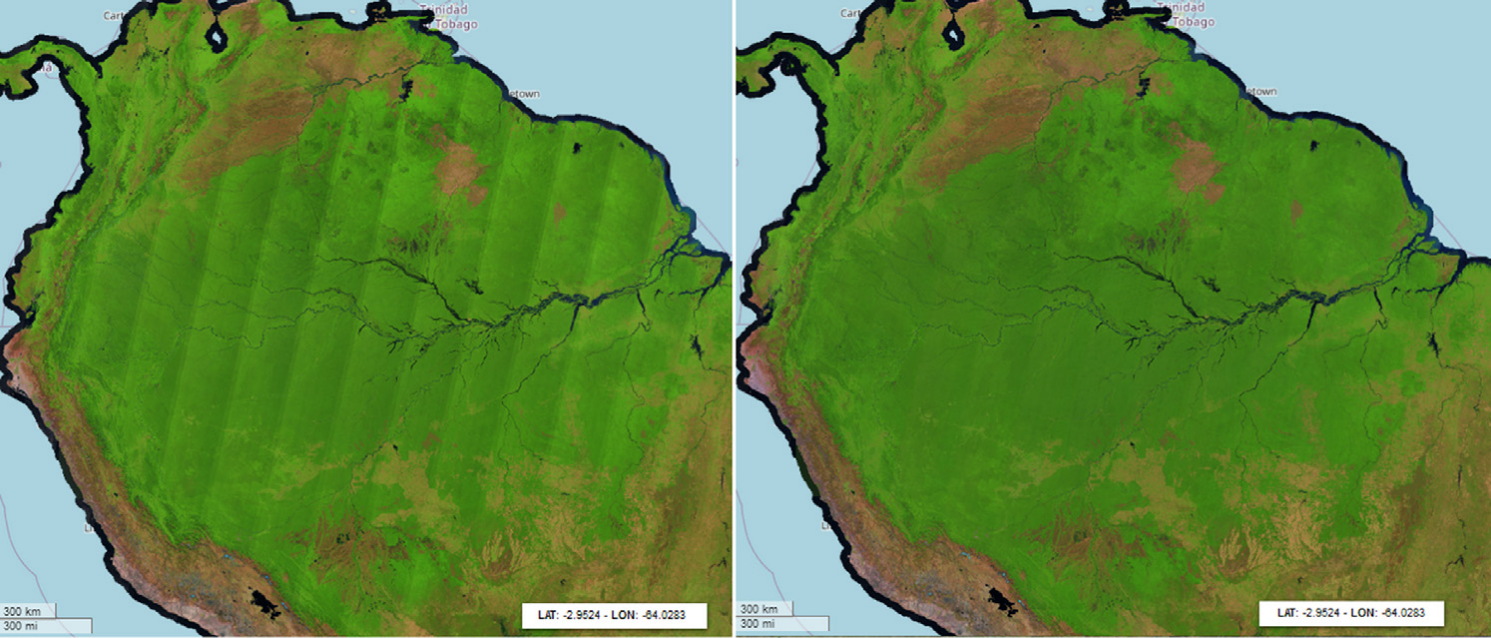
South America: improvements introduced by the orbit correction can be better seen at continental scale by comparing the 2018 (left) and 2020 (right) composites. Both products are visualized in false colour composite (SWIR1, NIR, RED).

Considering the above-mentioned limitation, the 2020 composite has been produced by applying a post processing orbit normalization using the IMPACT Toolbox [19] consisting of a multiplicative gradient ranging, over humid forest, from -12% to 0% (west-east) for the RED, NIR and SWIR1 bands; the effect of the gradient is visible when comparing 2018 and 2020 composites as reported on Fig. 15.

### 2.4 Indication for potential change between 2015–2017 / 2018 / 2019 / 2020

Potential change layers are generated 'on the fly' by the web-platform based on the spectral difference of the SWIR1 bands of two selected yearly composite (Y1, Y2) and rendered in a in a false colour combination as follows R: SWIR1^Y2^, G: SWIR1^Y1^, B: SWIR1^Y2^. Purple and green colours correspond to an increase (e.g., due to soil component) and a decrease (absorption e.g., due to vegetation growth or water) in the SWIR1 band, respectively. These layers may serve as a ready-to-use source of information to identify potential land cover changes, to be then confirmed by visual inspection of the reference annual composites or by supplementary materials.

**Fig. 16:**
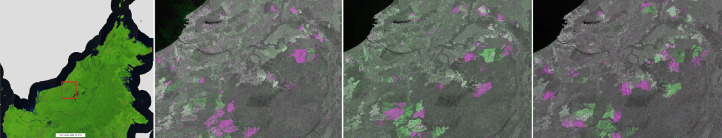
Malaysia. From left: 2020 composite in false colour (SWIR1, NIR, RED), change in 2018, 2019 and 2020. Vegetation loss (purple) and gain (green) as depicted by the false colour combination SWIR1^Y2^, SWIR1^Y1^, SWIR1^Y2^. This region is affected by a vast deforestation propagating from the edge into the forest.

**Fig. 17:**
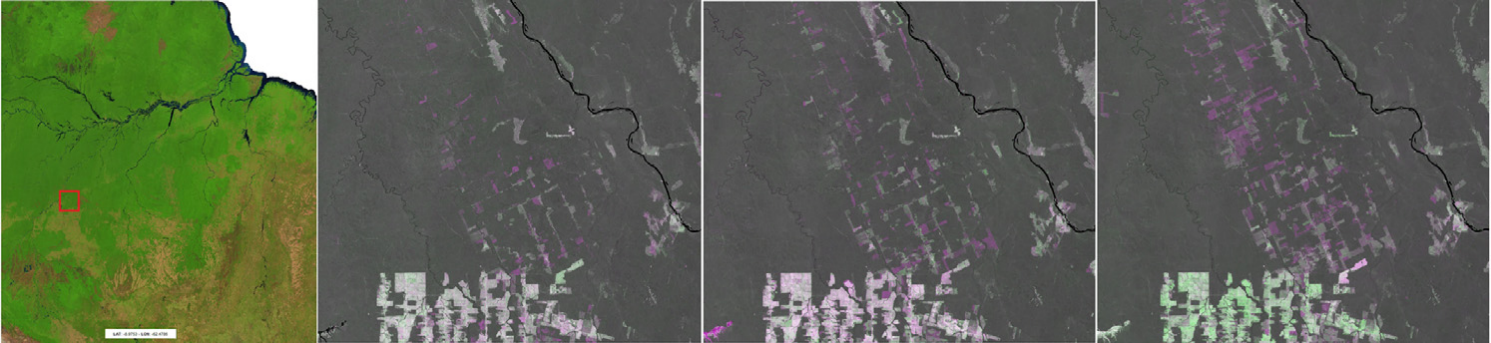
Rondonia, Brazil. From left: 2020 composite in false colour (SWIR1, NIR, RED), change in 2018, 2019 and 2020. Vegetation loss (purple) and gain (green) as depicted by the false colour combination SWIR1^Y2^, SWIR1^Y1^, SWIR1^Y2^. Deforestation is relatively big and follows a geometric pattern.

**Fig. 18:**
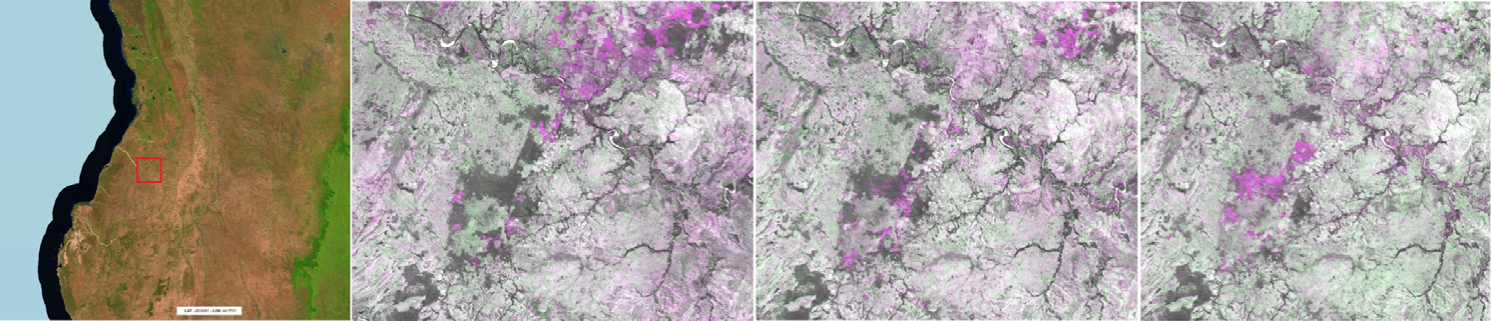
Madagascar. From left: 2020 composite in false colour (SWIR1, NIR, RED), change in 2018, 2019 and 2020. Vegetation loss (purple) and gain (green) as depicted by the false colour combination SWIR1^Y2^, SWIR1^Y1^, SWIR1^Y2^. This area is characterized by scattered, small-scale deforestation.

**Fig. 19:**
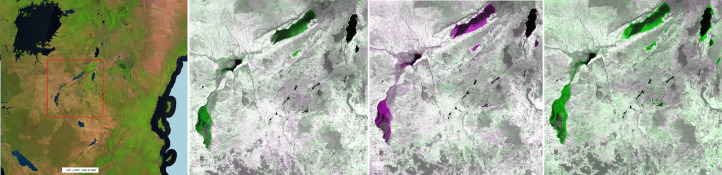
Tanzania. From left: 2020 composite in false colour (SWIR1, NIR, RED), change in 2018, 2019 and 2020. Water loss (purple) and gain (green) as depicted by the false colour combination SWIR1^Y2^, SWIR1^Y1^, SWIR1^Y2^. Year 2019 shows a remarkable shrinking of the main lakes while in 2020 there is a general abundance of water.

Examples of land cover changes as derived from the proposed annual composites, are reported on Figs. 16–19 covering locations in Malaysia, Brazil, Madagascar and Tanzania, respectively. Vegetation loss (purple) is quite evident in Malaysia, characterized by vast deforested areas followed by re-greening (green) in the subsequent years. Less pronounced but still evident, the geometric deforestation patches in Brazil while a more fragmented and small-scale pattern is observable in Madagascar. Apparently, in Tanzania, the layers show evident changes around lakes; a closer look confirms the drop and the increase of the SWIR1 band across the years caused by the inter-annual water dynamics. Being based on the change of the SWIR1 intensity over time, the potential change layer per-se is not capable of discriminating soil to vegetation transitions from soil to water and vice versa.

## Ethics Statement

Not applicable.

## CRediT Author Statement

**D. Simonetti** was the principal investigator of this study, he designed and developed the cloud mask and compositing algorithm in GEE, downloaded and equalized individual satellite swaths, organized and optimized data for dissemination and developed the server back-end. The manuscript was prepared by **D. Simonetti** with the contributions of **U. Pimple** and **A. Marelli**.

**A. Marelli** developed the front-end of the Sentinel-2 cloud-free explorer; **A. Langner** contributed to the analysis of the results providing useful feedback on the products in the framework of the ReCaREDD (Reinforcement of Capacities for REDD+) and REDDCopernicus projects.

## Declaration of Competing Interest

The authors declare that they have no financial interests or personal relationships that could have appeared to influence the work reported in this paper.
